# Cross-cultural adaptation, validation and psychometric evaluation of the International Hip Outcome Tool 12 (iHOT_12_) to Hebrew

**DOI:** 10.1186/s12955-023-02203-0

**Published:** 2023-11-21

**Authors:** Yael Steinfeld-Mass, Noa Ben-Ami, Itamar Botser, David Morgenstern, Aharon S. Finestone

**Affiliations:** 1 Faculty of Medicine, Tel Aviv University, Ramat Aviv, P.O.B 39040, 69978 Tel Aviv, Israel; 2 Department of Physiotherapy, Ariel University, Ariel, Israel; 3 Assuta Medical Center, Ramat HaHayal, Tel-Aviv, Israel; 4 Departement of Orthopaedic Surgery, Rambam Health Care Campus affiliated to the Bruce Rappaport Faculty of Medicine, Technion, Haifa, Israel; 5 Departement of Orthopaedic Surgery, Shamir (Assaf HaRofeh) Medical Center, Beer Yaakov, Israel

**Keywords:** Patient reported outcome measures, Hip pain, International hip outcome tool 12 (iHOT_12_), Validity, Reliability, Hebrew

## Abstract

**Background:**

The “International Hip Outcome Tool 12” (iHOT_12_) is a self-administered patient-reported outcome tool for measuring health-related quality of life and physical functioning in young and active patients with hip pathology. Since the iHOT_12_ has become widely used, we sought to translate and validate it for Hebrew-speaking populations. The aims of this study were: (1) To translate and culturally adapt the iHOT_12_ into Hebrew using established guidelines. (2) To test the new Hebrew version for validity, and (3) reliability.

**Methods:**

The iHOT_12_ was translated and culturally adapted from English to Hebrew (iHOT_12_-H) according to the COSAMIN guidelines. For validity, the iHOT_12_-H and Western Ontario and McMaster universities osteoarthritis index (WOMAC) were completed by 200 patients with hip pathology. Exploratory factor analysis was used to assess structural validity. Subsequently, 51 patients repeated the iHOT_12_-H within a 2-week interval. Intraclass Correlation Coefficient (ICC), Cronbach alpha, and Standard Error of Measurement (SEM) were calculated to assess reliability.

**Results:**

Construct validity: iHOT_12_-H correlated strongly to the WOMAC scores (*r* = -0.82, *P* < 0.001, Spearman). Factor analysis revealed a two-factor structure. Cronbach’s alpha was 0.953 confirming internal consistency to be highly satisfactory. Test–retest correlation of the iHOT_12_-H was excellent with an ICC = 0.956 (95% CI 0.924–0.974). There was no floor or ceiling effect.

**Conclusion:**

The iHOT_12_ Hebrew version has excellent reliability, good construct validity and can be used as a measurement tool for physical functioning and quality of life in young, physically active patients with hip pathology. This study will serve Israeli researchers in evaluating treatment effectiveness for these patients. Moreover, it will also enable multinational cooperation in the study of hip pathology.

**Supplementary Information:**

The online version contains supplementary material available at 10.1186/s12955-023-02203-0.

## Highlights

• The International Hip Outcome Tool 12 was translated into Hebrew (iHOT_12_-H).

• The iHOT_12_-H showed comparable psychometric properties to the original version.

• The iHOT_12_-H is a valid and reliable questionnaire for people with hip-related pain.

**Supplementary Information:**

The online version contains supplementary material available at 10.1186/s12955-023-02203-0.

## Background

Hip-related pain is known to be a major contributor to years lived with disability [1], causing functional loss and low patient-reported outcomes scores in many young and active people [2, 3]. In recent years, as hip arthroscopic technology has evolved, there has been a significant increase in diagnosis and surgical management for different hip pathologies in younger populations around the world [4, 5].

To assess the impact of hip pathologies and to measure the effect of hip arthroscopic surgery and conservative management, it is important to use health related patient reported outcome measurements (HR-PROM’s) [6]. HR-PROM’s are questionnaires completed by patients to measure their subjective perception of their health, pain and function about a specific condition. Thus, they have been recognized as important tools in assessing conservative and surgical management effects on different Musculoskeletal conditions [6].

Several HR-PROM’S have been developed over the years to evaluate different lower limb and hip-related disorders [7, 8]. However, most of these questionnaires were originally developed to assess older patients with osteoarthritis or undergoing hip arthroplasty [7–10]. As young and active patients undergoing hip arthroscopy have different expectations and goals, Throborg et al. recommended to reconsider their applicability for this population [11, 12]. The 33-item International Hip Outcome Tool (iHOT_33_) developed by Mohtadi et al. has addressed this limitation as it was developed to assess young active patients with hip joint disorders [13]. This questionnaire showed high validity and reliability in measuring physical functioning and quality of life among young, physically active patients with hip-related pain. Based on the iHOT_33_, Griffin et al. [14] developed a shorter version (iHOT_12_), which has proved to have good validity, reliability, and responsiveness to change [14]. Due to its high psychometric properties, the iHOT_12_ has been translated into many languages, including Portuguese [15], Swedish [16], Dutch [17], German [18], Japanese [19], Turkish [20, 21], Greek [22], and French [23]. The iHOT_12_ has not yet been translated and culturally adapted into Hebrew. The aims of this study were to: 1) to translate the English version of the iHOT_12_ into Hebrew and to adapt it culturally to a Hebrew speaking population; 2) to test the new Hebrew version for validity and reliability.

## Methods

### Study design 

The translation to Hebrew and the validation process of the translated iHOT_12_ were conducted between September 2020 to December 2021. The process consisted of two steps: 1) translation and cross-cultural adaptation of the English iHOT_12_ into Hebrew; 2) evaluation of the psychometric properties of the iHOT_12_-Hebrew version (iHOT_12_-H): internal consistency, test–retest reliability, standard error of measurement (SEM), floor and ceiling effects, and construct validity of the iHOT_12_-H with the Western Ontario and McMaster universities Osteoarthritis index (WOMAC). The study was reviewed and approved by the Medical Ethical Committee of Assuta Medical Center (23.8.2020/0007–20-ASMC)”.

### Translation of the iHOT_12_

The translation was performed with the permission of the original author of the iHOT_12_ [14]. The iHOT_12_ was translated into Hebrew and culturally adapted according to the Consensus-based Standards for the selection of health measurement instruments (COSMIN) guidelines for best practice in questionnaire translation including five stages [24]. In stage 1 (translation), the English version of the iHOT_12_ was translated to Hebrew by two Hebrew native speakers (two independent versions) who were also fluent in English; an orthopedic surgeon (with over 20 years of experience) and a physiotherapist with a PHD degree (with more than 15 years of experience). A third translator was a professional translator, meeting the need for a translator who is not a health provider, naïve to the questionnaire’s concepts. Backward translation was performed by two bilingual native English speakers, who independently translated the Hebrew version of the iHOT_12_ back into English. Both were naïve to the questionnaire’s concepts. An expert committee consisted of an orthopedic doctor specializing in musculoskeletal conditions (and their measurement) in pain population research (MD, MHA), a physiotherapist and pain researcher experienced with a cross-cultural adaptation of questionnaires (PhD, PT), and a physiotherapist with over 20 years’ experience in the public and private sector. Subsequently, the investigator and the same experts team came to an agreement on the pre-final version of the iHOT_12_-H.

The pre-final version of the iHOT_12_-H was tested on a group of patients with various hip pathologies (*N* = 30). As no changes were found necessary, the pre-final version was chosen as the final version of the iHOT_12_-H (Additional file 1).

### Participants 

Patients attending hip clinics of the 3^rd^ and 4^th^ authors were asked to participate in the study. Inclusion criteria were: men and women, between 18 and 60 years of age, who suffered from hip pain. Following informed consent, they completed the iHOT_12_-H and WOMAC questionnaires.

### Measurement instruments

#### The iHOT_12_-H

The English iHOT_12_ is a valid and reliable disease-specific questionnaire that measures physical function and health-related quality of life in a younger patient population with hip pathology [14]. The iHOT_12_ consists of 12 questions with a 100-mm visual analog scale. Each question has equal weight and is scored between 0 (maximum limitation) and 100 (full function). The final score is calculated as the mean of all questions ranging from 0 to 100. Higher scores reflect better physical functioning and better health-related quality of life [14]. Missing values are ignored, and the score is the mean of the existing values. The validation evaluation of the iHOT_12_ showed good agreement between the iHOT_12_ to the iHOT_33_, with regression analysis showing that the iHOT_12_ accounted for 95.9% (95% CI, 95.0% to 96.8%) of the variation in the iHOT_33_. The test–retest reliability was found to be good, with an intraclass correlation coefficient of 0.89 (95% boot-strapped CI, 0.83 to 0.93) [14].

#### The WOMAC

The WOMAC is a 24-item questionnaire. A valid and reliable Hebrew version is available [25]. Subjects rate their level of suffering using a visual analogue scale (10 cm VAS) where 0 represents no suffering while 10 represents high level of suffering. The results were standardized to a scale of 0 to 100 and the final scores were the mean of the 24 items. The validation evaluation of the WOMAC showed significant correlations (*p* < 0.01) between the WOMAC items and visual analog scale (VAS) of pain and handicap. The test–retest reliability Pearson’s correlation coefficients for the WOMAC items ranged from 0.55 to 0.78 (*p* < 0.01), and the Cronbach’s alpha ranged between 0.97 (time 1) and 0.98 (time 2) [25].

### Procedures

#### Validity

Construct validity is the extent to which the results of the translated questionnaire correlate with results of other questionnaires that measure the same construct [24]. In this study, we evaluated the magnitude of relationships between the iHOT_12_-H and the WOMAC questionnaires.

#### Reliability

To describe reliability of the iHOT_12_-H we assessed internal consistency, measurement error, and test–retest reliability. For test–retest reliability, 51 patients completed the iHOT_12_-H twice within a 2-week interval. Participants were also asked whether they had improved or worsened over the past two weeks and were included only if symptoms had not changed. This time interval was considered adequate to prevent the patients from remembering their answers (“recall bias”), and short enough to ensure that clinical change had not occurred.

#### Sample size

For validity, the input parameters were as follows: assuming a modest effect size of 0.3, α = 0.05 and β = 0.9, considering loss of 10% subjects, the total sample size recommended was at least 126 patients. For test–retest reliability we assumed that the intraclass correlation coefficient (ICC) score will be more than 0.8, with a power of 0.8, the sample size recommended was of 51 patients.

### Statistical analysis

Statistical analysis was performed using the IBM SPSS Statistics software, version 28.0 (IBM Corporation, Armonk, NY). Normal distribution of all data was assessed by the Kolmogorov–Smirnov test. Patient characteristics were analyzed by means of descriptive statistics. A *P* value less than 0.05 (*P* < 0.05) was used to indicate statistical significance.

#### Reliability

Reliability is the degree to which the measurement is free from measurement error [24]. To evaluate reliability, internal consistency, test–retest reliability, and measurement error were calculated [26].

#### Internal consistency

For internal consistency we calculated Cronbach’s alpha, with the following ratings: weak correlation: 0–0.50, medium: 0.50–0.75, very good: 0.75–0.90, and excellent: > 0.90 [26].

#### Test–retest reliability

For test–retest reliability we used intraclass correlation coefficient (ICC), implementing the two-way mixed effect test–retest absolute agreement method. The ICC values were as follows: poor: < 0.40, fair: 0.40–0.59, good: 0.60–0.74, and excellent: 0.75–1.00 [27]. Interpretability and repeatability refer to the degree to which one can assign qualitative meaning to quantitative scores [24]. It was determined by calculating floor and ceiling effects, which are present if more than 15% of respondents have the lowest or highest possible score [26].

#### Measurement error

The standard error of measurement (SEM) was calculated using the formula $\mathrm{SEM}=\mathrm{SD}\times \sqrt{1-\mathrm{ICC}}$, where SD = standard deviation [26].

#### Validity

To validate the Hebrew translation of the iHOT_12_, it was compared to the WOMAC scores using the Spearman correlation coefficient (not all outcomes were normally distributed). The accepted grading criteria were used: 0 to 0.39 weak correlation, 0.40 to 0.59 medium correlation:, and 0.6 to 1.0 strong correlation [28].

#### Factor analysis

The structural validity of the iHOT_12_-H questionnaire was examined using exploratory factor analysis (EFA). This analysis employed a maximum likelihood extraction method with varimax rotation to identify the latent factor structure of the questionnaire. Initially, to assess the appropriateness of the data to factor analysis, Bartlett’s sphericity and the Kaiser–Meyer–Oklin (KMO) tests were applied. Factors with eigenvalues exceeding 1 and items with factor loadings of 0.40 or higher were retained. Additionally, a scree plot was used to determine the optimal number of factors in the questionnaire [29].

## Results

### Participants 

The final data analysis of the cross-cultural translation, adaptation, and validation research of the iHOT_12_-H provided a total sample size of 200 patients (110 females, 55%). The mean age was 39.8 with standard deviation (SD) of 13.0. Mean scores and standard deviation are shown in Table 1.

**Table 1:** Demographic data and diagnostic related scores of all participants (*n* = 200)

Variable	Mean ± SD	Range
Age	39.8 ± 13.0	18 – 60
Gender, N (%)		
Female	110 (55%)	
Male	90 (45%)	
iHOT_12_-H	49.6 ± 23.0 (Median: 50.8)	1.9 – 96.3 (IQR: 36.3)
WOMAC	34.6 ± 24.5 (Median: 29.1)	0.0 – 90.8 (IQR: 40.1)

*Abbreviations*: *iHOT*_*12*_ International Hip Outcome Tool, *IQR* Interquartile Range, *SD* Standard Deviation, *WOMAC* Western Ontario and McMaster Universities Osteoarthritis Index

### Translation and cross-cultural adaptation

During the forward/backward translations, we found only minor linguistic differences: answers for items 1 and 7 (“extreme pain”), and item 4 (“grinding” and “catching”). The answer for items 1 and 7 of “extreme pain” was different between the three forward versions “significant pain”. After discussing this in the expert committee, we agreed that the translation of “significant pain” suited the source better. The translation proposed for item 4 was challenging because not all patients understood the words “grinding” and “catching”. However, during examination of the pre-final version with patients suffering from hip pain it seemed that people without those symptoms, were those who didn’t understand the translated terms of “grinding” and “catching”, but subjects with those feelings immediately understood what the terms meant.

### iHOT_12_-H psychometric properties: test–retest reliability 

Internal consistency for the iHOT_12_-H was excellent with Cronbach’s α = 0.953. The final test–retest reliability sample included 51 participants. However, for the ICC calculation of question number 9, only 46 participants were included. This question (“how much trouble do you have with sexual activity because of your hip”) was marked “not relevant for me” by 5 participants. Thus, those were excluded from the ICC calculation. The iHOT_12_-H translated version demonstrated excellent test–retest reliability with ICC = 0.956, 95% Confidence interval (CI) (0.924–0.974). Standard Error of Measurement (SEM) was calculated using the ICC values as described elsewhere [26]. Each item’s ICC and SEM are shown in Table 2. No floor or ceiling effects were found.

**Table 2:** Intraclass correlation coefficient (ICC) with 95% confidence intervals (CI) and standard error of measurement (SEM) for test–retest reliability of the translated iHOT_12_ (*n* = 51)

	**ICC**	**95% CI**	**SEM**
**Total score**	0.956	0.924–0.974	5.151
1:iHOT_1_	0.884	0.805–0.932	9.733
2:iHOT_2_	0.789	0.658–0.874	13.975
3:iHOT_3_	0.777	0.640–0.866	15.090
4:iHOT_4_	0.812	0.692–0.888	14.637
5:iHOT_5_	0.874	0.790–0.926	11.367
6:iHOT_6_	0.874	0.790–0.926	10.974
7:iHOT_7_	0.774	0.635–0.865	14.295
8:iHOT_8_	0.903	0.834–0.944	9.861
9:iHOT_9_	0.843	0.733–0.910	12.844
10:iHOT_10_	0.783	0.648–0.870	14.704
11:iHOT_11_	0.901	0.833–0.942	7.958
12:iHOT_12_	0.919	0.862–0.953	8.798

*Abbreviations*: *CI* Confidence Intervals, *ICC* Intraclass Correlation Coefficient, *SEM* Standard Error of Measurement

### Construct validity

**Fig. 1:**
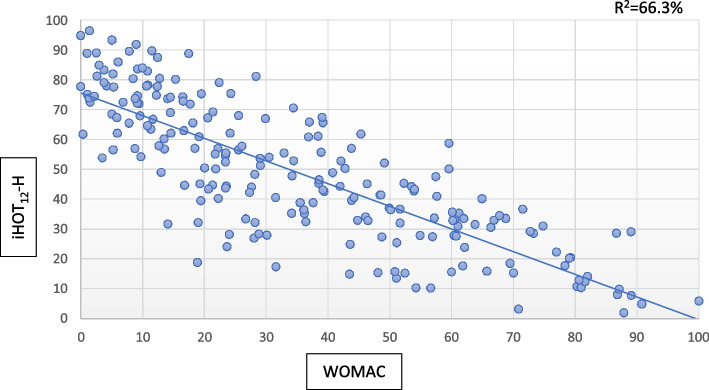
Relation between WOMAC and iHOT_12_-H for validation data (*n* = 200)

Construct validity was evaluated with correlation analysis between the mean results of the iHOT_12_-H and the WOMAC scores. We used Spearman’s correlation coefficients as most of the questionnaires’ total scores were non-normally distributed. We found a good negative correlation of *r* = -0.82 (*P* < 0.001) between the iHOT_12_-H and the WOMAC scores (these scales are orientated in opposite directions, Fig. 1).

### Factor analysis

**Fig. 2:**
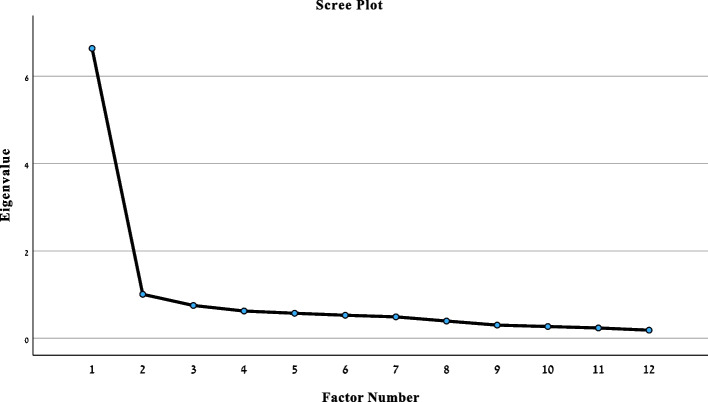
Scree plot indicating factor loading for iHOT_12_-H

The Bartlett test of sphericity yielded significant results (Chi square = 1217.73, *p* < 0.001), affirming the suitability of the data for factor analysis. Additionally, the KMO measure of sampling adequacy stood at 0.92, indicating the dataset’s appropriateness for this analysis. Through the examination, two distinct factors emerged, each with eigenvalues surpassing 1 and item factor loadings ≥ 0.40. Specifically, the first factor accounted for 30.2% of the variance, while the second factor explained 26.5% (with eigenvalues of 3.6 and 3.2, respectively). This two-factor structure was further supported by the scree plot (Fig. 2). Detailed factor loadings can be found in Table 3.

**Table 3:** Factor loading based on maximum likelihood with varimax rotation for the iHOT_12_-H

Items	Factor 1	Factor 2
iHOT_1_	0.522	
iHOT_2_	0.640	
iHOT_3_	0.727	
iHOT_4_	0.461	
iHOT_5_	0.744	
iHOT_6_		0.490
iHOT_7_	0.566	
iHOT_8_	0.743	
iHOT_9_	0.564	
iHOT_10_		0.776
iHOT_11_		0.591
iHOT_12_		0.846

## Discussion

This study aimed to translate and culturally adapt the iHOT_12_ to Hebrew and test its psychometric properties. From this study it can be concluded that the translation procedure of the English iHOT_12_ was successful. The results of this study show that the iHOT_12_-H is a reliable, internally consistent, and valid measurement tool to assess physical functioning and quality of life in an Israeli population of young, physically active individuals between 18 to 60 years of age with hip-related pain.

Hip related pain has become one of the most commonly diagnosed musculoskeletal conditions in young and active adults, leading to increased hip arthroscopic surgery rates. However, until recently, there has been a lack of standardized patient-reported outcome measures for this specific population. The iHOT_33_, and consequently the iHOT_12_, have addressed this limitation. The favorable psychometric characteristics of the iHOT_12_ and the relatively short time for application enables it to be used in research as well as in daily clinical practice. Furthermore, a few systematic reviews questioning which patients reported outcome measures are most responsive in this patient population further reinforce the validity of the iHOT_12_ in assessing outcome for treatment of young and active patients [9, 30, 31].

### Study population

Our demographic data included men and women with an average age of 39.8 ± 13.0. Thus, they are comparable with the average age used in the original study of the development of iHOT_12_ conducted by Griffin et al. [14]. In our study, to have a more heterogenous patient sample, we did not preselect patients according to their diagnosis or intended treatment. Two of the previous translation and validation studies of the iHOT_12_ evaluated only patients with Femoro-acetabular impingement syndrome (FAIS) [16, 19]. This may have negatively affected the external validity of their study.

### Reliability

The overall assessment of the iHOT_12_-H yielded remarkably high values. These results prove the quality of the iHOT_12_-H version and confirm the results of previous validation studies on the iHOT_12_ [9, 13–20, 22, 23]. The iHOT_12_-H showed good internal consistency, with a Cronbach alpha of 0.953. This result is comparable with the Cronbach alpha values evaluated in prior studies: Swedish (α = 0.89) [16], Dutch (α = 0.96) [17], German (α = 0.94) [18], Japanese (α = 0.90) [19], Turkish (α = 0.93) [20], and Greek (α = 0.92) [22] versions of the iHOT_12_. The fact that in the present study we found a Cronbach alpha higher than 0.95, may indicate that the items in this questionnaire are almost the same construct [24]. Future studies may look at the possibility of removing some of the items of the iHOT_12_.

The iHOT_12_-H showed excellent test–retest reliability, with an ICC of 0.956 (95% CI 0.924–0.974), which is comparable with the ICC of the English (ICC = 0.89) [14], Swedish (ICC = 0.88) [16], Dutch (ICC = 0.93) [17], German (ICC = 0.94) [18], Japanese (ICC = 0.89) [19], Turkish (ICC = 0.93) [20] and Greek (ICC = 0.98) [22] versions. The French version of the iHOT_12_ showed lower values of ICC (ICC = 0.84) [23], but these values are still categorized as good test–retest reliability [27].

### Validity

For the evaluation of construct validity, we chose the WOMAC questionnaire, as it was hip-specific and validated questionnaire in the Hebrew language [25]. We found strong correlation between the iHOT_12_-H and the WOMAC score (*r* = -0.82, *P* < 0.001). To our knowledge, these relationships have been investigated previously only by Attila et al. [20], who found a similar correlation between the iHOT_12_-T and the WOMAC score (*r* = 0.815, *P* < 0.001) [20]. All other studies evaluating the validity of the iHOT_12_ following a procedure of translation used a variability of PROM forms as the gold standard [14, 16–19, 22, 23]. Li et al. [32] evaluated the correlation between the iHOT_33_ and the WOMAC score and found similar correlation coefficient (*r* = 0.812) to our results.

### Factor analysis

The factor analysis of the iHOT_12_-H revealed a two-factor structure: Factor-1 (items 1–5, 7–9) refers to “symptoms and functionality”, while Factor-2 (items 6, and 10–12) refers to “hip related concerns”. The original English version of the iHOT_12_ has a single factor structure [14]. Likewise, the Dutch and one of the Turkish versions reveled a one-factor structure [17, 20]. However, the Swedish version showed two factors, but with different factor loadings than ours: Factor-1 “Function and symptoms” (items 2–5, 8, 9) and Factor-2 “pain and concern/destruction” (items 1, 6, 7, 10–12) [16]. Another study of a Greak version showed two factors quite similar to our results: factor-1 “symptoms and functionality” (items 1–9) and Factor-2 “hip disorder-related concerns” (items 10–12) [22]. Interestingly, we found a second study validating a Turkish version who revealed 3 factors: “Symptom and functional limitations” (items 1–4), “Social, emotional and lifestyle” (items 8–12), and “Sports and recreational activities” (items 6, 7, 11) [21]. Factor-model variations may result from cross-cultural factors, or from age-related quality of life concerns, as the mean age of the studied population varies among studies [17, 20–22]. For the Portuguese, German, Japanese and French versions, no factor analysis has been conducted [15, 18, 19, 23]. 

### Limitations

Despite very good results concerning validity and reliability, there are a few limitations in this study. First, we included patients with different levels of activity but we did not evaluate the exact activity level of our patients using the Tegner Activity Scale as was evaluated in some of the previous studies [17–19]. Thus, future research comparing between different levels of activity of patients is therefore necessary to determine whether the iHOT_12_ is applicable to such a variety of patients. Secondly, the study sample included patients with a variety of hip pathologies. Including a heterogenous population may also increase the external validity of this study. Thirdly, since the iHOT_33_ questionnaire has not been officially translated into the Hebrew language we were unable to assess the criterion-related validity of iHOT_12_-H [24]. Finally, responsiveness was not determined in this study, therefore we could not evaluate the exact minimal important change values. Additional research is needed to determine whether the iHOT_12_-H is a responsive instrument as was shown in previous studies [16, 18, 19, 22]. Further prospective studies are needed to assess the clinical impact of iHOT_12_ on patients with hip related pain who underwent conservative management or surgical treatment. Such studies will advance our understanding of the therapeutic processes among those patients and will provide benefits both in clinical practice and in research.

## Conclusions

The iHOT_12_-H is a reliable and valid measurement tool for measuring physical functioning and quality of life in young, physically active patients with hip related pain. This is extremely important, as previous tools are less suitable for this young population. We believe that this HR-PROM is beneficial in assessing the condition of Israeli patients with hip related pain.

### Supplementary Information

**Additional file 1.** The Hebrew version of the iHOT_12_.

## Data Availability

This study contains all of the study data related to these findings. Requests for mor information about data sharing should be directed to the correspondence author via ys3@mail.tau.ac.il.

## Abbreviations

CI: Confidence interval
COSMIN: Consensus-based Standards for the selection of health measurement instruments
EFA: Exploratory factor analysis
FAIS: Femoro-acetabular impingement syndrome
HR-PROM’s: Health related patient reported outcome measurements
ICC: Intraclass correlation coefficient
iHOT_12_: International hip outcome tool 12
KMO: Kaiser–Meyer–Oklin
SD: Standard deviation
SEM: Standard error of measurement
VAS: Visual analogue scale
WOMAC: Western Ontario and McMaster universities Osteoarthritis index
