# Supplementary material for: Cross-cultural adaptation, validation and psychometric evaluation of the International Hip Outcome Tool 12 (iHOT12) to Hebrew
Source: Health Qual Life Outcomes. 2023 Nov 21;21:127. doi: 10.1186/s12955-023-02203-0 (PMC10662524; doi:10.1186/s12955-023-02203-0)
Supplement: Supplementary file 1 — Additional file 1. The Hebrew version of the iHOT12. [file 12955_2023_2203_MOESM1_ESM.pdf]

Translated version of the i-HOT$_{12}$ to Hebrew (iHOT$_{12}$-H)

iHOT[12]

שאלון תפקוד מפרק הירך הבין-לאומי לאנשים צעירים

אנא סמן איזו ירך מציקה לך יותר, והתיחס לירך זו בכל השאלון:

☐ שמאל

☐ ימין

שם פרטי: __________ משפחה: ______________

מס' ת"ז: ________________ מגדר: ☐ נקבה

תאריך לידה: ___ / ____ / ___ ☐ זכר

תאריך מילוי השאלון: ___ / _____ / _____

טיפ: אם אינך מבצע פעילות מסוימות, דמיין לעצמך איך הירך היה מרגיש אילו היית צריך לעשות זאת.

הוראות:

- ✓ השאלון מנוסח בלשון זכר לנשים וגברים כאחד.
- ✓ השאלות הבאות מופנות לבעיות שייתכן ואתה חש במפרק הירך שלך, כיצד הן משפיעות על חייך, והרגשות שאתה חש בגלל בעיות אלו.
- ✓ אנא ציין את מידת החומרה על ידי סימון קו אלכסוני על גבי הקו המופיע מתחת לכל שאלה.
  - ➢ סימון בחלק **הימני** של הקו, משמעו שאתה מרגיש **מוגבל משמעותית**.

לדוגמא:

מוגבל במידה משמעותית |/——————————————————| ללא כל הגבלה

  - ➢ סימון בחלק **השמאלי** של הקו, משמעו שאתה **לא חושב שיש לך כל בעיה עם מפרק הירך**.

לדוגמא:

מוגבל במידה משמעותית |——————————————————/| ללא כל הגבלה

  - ➢ סימון במרכז הקו זה מצביע על כך שאתה מוגבל במידה בינונית. במילים אחרות, בין הקצוות של "מוגבל במידה משמעותית" לבין "ללא כל הגבלה".

- ✓ התייחס בתשובתך לתחושתך **מהחודש** האחרון.

ש1: באופן כללי, כמה כאב אתה חשה במפרק הירך/מפשעה?

כאב קיצוני |——————————————————| ללא כאב כלל

ש2: כמה קשה לך להתרומם מעלה מהרצפה או לרדת לרצפה?

קשה ביותר |——————————————————| לא קשה כלל

ש3: כמה קשה לך ללכת מרחקים ארוכים?

קשה ביותר |——————————————————| לא קשה כלל

ש4: כמה מפריעה לך תחושה של "חיכוך", "תפיסות" או "קליקים" במפרק הירך שלך?

מפעירה מאד |——————————————————| לא מפריעה כלל

ש5: כמה מפריע לך לבצע דחיפה, משיכה, או הרמה של חפצים כבדים?

מפריע מאד |———————| לא מפריע כלל

ש6: כמה אתה מודאג מביצוע שינוי כיוון מהיר במהלך פעילות ספורטיבית או פנאי?

מודאג מאד |———————| לא מודאג כלל

ש7: כמה כאב אתה חש במפרק הירך לאחר פעילות?

כאב קיצוני |———————| ללא כאב כלל

ש8: כמה אתה מודאג מהרמה או מנשיאה של ילדים בשל מפרק הירך?

מודאג מאד |———————| לא מודאג כלל

ש9: כמה בעייתי מבחינתך לקיים יחסי מין לאור הבעיה במפרק הירך? ☐ סמן בריבוע אם לא רלוונטי

בעייתי מאד |———————| לא בעייתי כלל

ש10: כמה אחוז מהזמן אתה חושב על המגבלה במפרק הירך?

כל הזמן |———————| לא חושב כלל

ש11: עד כמה אתה מודאג מיכולתך לשמור על רמת הכושר אליה אתה שואף?

מודאג מאד |———————| לא מודאג כלל

ש12: עד כמה הבעיה במפרק הירך מסיחה את דעתך מדברים אחרים?

מסיחה מאד |———————| לא מסיחה כלל
